# Fractional Flow Reserve-Guided Stent Optimisation in Focal and Diffuse Coronary Artery Disease

**DOI:** 10.3390/diagnostics13152612

**Published:** 2023-08-07

**Authors:** Hirofumi Ohashi, Damien Collison, Takuya Mizukami, Matthaios Didagelos, Koshiro Sakai, Muhammad Aetesam-ur-Rahman, Daniel Munhoz, Peter McCartney, Thomas J. Ford, Mitchell Lindsay, Aadil Shaukat, Paul Rocchiccioli, Richard Brogan, Stuart Watkins, Margaret McEntegart, Richard Good, Keith Robertson, Patrick O’Boyle, Andrew Davie, Adnan Khan, Stuart Hood, Hany Eteiba, Tetsuya Amano, Jeroen Sonck, Colin Berry, Bernard De Bruyne, Keith G. Oldroyd, Carlos Collet

**Affiliations:** 1 Cardiovascular Center Aalst, OLV Clinic, 9300 Aalst, Belgium; hirofumiohashi@coreaalst.com (H.O.); koshirosakai@coreaalst.com (K.S.); danielmunhoz@coreaalst.com (D.M.); jeroensonck@coreaalst.com (J.S.); bernard.de.bruyne@olvz-aalst.be (B.D.B.); carloscollet@gmail.com (C.C.); 2 Department of Cardiology, Aichi Medical University, Nagakute 480-1195, Japan; amanotaha@yahoo.co.jp; 3 West of Scotland Regional Heart & Lung Centre, Golden Jubilee National Hospital, Clydebank G81 4DY, UK; dgcollison@gmail.com (D.C.); manthosdid@yahoo.gr (M.D.); m.aetesam-ur-rahman@nhs.net (M.A.-u.-R.); peter.mccartney@glasgow.ac.uk (P.M.); mitchell.lindsay@gjnh.scot.nhs.uk (M.L.); aadil.shaukat@nhs.scot (A.S.); paul.rocchiccioli@gjnh.scot.nhs.uk (P.R.); richard.brogan@gjnh.scot.nhs.uk (R.B.); stuart.watkins@gjnh.scot.nhs.uk (S.W.); margaret.mcentegart@gmail.com (M.M.); richard.good@gjnh.scot.nhs.uk (R.G.); keith.robertson@gjnh.scot.nhs.uk (K.R.); patrick.oboyle@hse.ie (P.O.); andrew.davie@ggc.scot.nhs.uk (A.D.); adnankhan@nhs.net (A.K.); stuart.hood@ggc.scot.nhs.uk (S.H.); hany.eteiba@glasgow.ac.uk (H.E.); colin.berry@glasgow.ac.uk (C.B.); kgoldroyd@gmail.com (K.G.O.); 4 Institute of Cardiovascular & Medical Sciences, University of Glasgow, Glasgow G12 8QQ, UK; tom.ford@health.nsw.gov.au; 5 Clinical Research Institute for Clinical Pharmacology and Therapeutics, Showa University, Tokyo 157-8577, Japan; 6 Department of Cardiovascular Medicine, Gifu Heart Center, Gifu 500-8384, Japan; 7 Department of Cardiology, Showa University Hospital, Tokyo 142-8666, Japan; 8 Department of Advanced Biomedical Sciences, University Federico II, 80138 Naples, Italy; 9 Faculty of Medicine, University of Newcastle, Central Coast Campus, Ourimbah, NSW 2258, Australia; 10 Department of Cardiology, Lausanne University Hospital, 1005 Lausanne, Switzerland

**Keywords:** coronary artery disease, fractional flow reserve, revascularisation, pullback pressure gradient, PCI optimisation

## Abstract

Assessing coronary physiology after stent implantation facilitates the optimisation of percutaneous coronary intervention (PCI). Coronary artery disease (CAD) patterns can be characterised by the pullback pressure gradient (PPG) index. The impact of focal vs. diffuse disease on physiology-guided incremental optimisation strategy (PIOS) is unknown. This is a sub-study of the TARGET-FFR randomized clinical trial (NCT03259815). The study protocol directed that optimisation be attempted for patients in the PIOS arm when post-PCI FFR was <0.90. Overall, 114 patients (*n* = 61 PIOS and 53 controls) with both pre-PCI fractional flow reserve (FFR) pullbacks and post-PCI FFR were included. A PPG ≥ 0.74 defined focal CAD. The PPG correlated significantly with post-PCI FFR (r = 0.43; 95% CI 0.26 to 0.57; *p*-value < 0.001) and normalised delta FFR (r = 0.49; 95% CI 0.34 to 0.62; *p*-value < 0.001). PIOS was more frequently applied to vessels with diffuse CAD (6% focal vs. 42% diffuse; *p*-value = 0.006). In patients randomized to PIOS, those with focal disease achieved higher post-PCI FFR than patients with diffuse CAD (0.93 ± 0.05 vs. 0.83 ± 0.07, *p* < 0.001). There was a significant interaction between CAD patterns and the randomisation arm for post-PCI FFR (*p*-value for interaction = 0.004). Physiology-guided stent optimisation was applied more frequently to vessels with diffuse disease; however, patients with focal CAD at baseline achieved higher post-PCI FFR.

## 1. Introduction

The optimisation of percutaneous coronary intervention (PCI) is advocated to reduce stent failure and improve patient outcomes [1]. After stent implantation, intravascular imaging can be used to optimise stent expansion, correct malapposition, and detect residual disease [2,3]. Fractional flow reserve (FFR) can also be used to optimise PCI by identifying physiologically sub-optimal results and guide additional intervention [4,5]. Performing additional interventions in segments of residual pressure losses translates into higher post-PCI FFR values, which may positively influence prognosis [4,6,7]. Recently, the TARGET-FFR (Trial of Angiography vs. Pressure-Ratio-Guided Enhancement Techniques-Fractional Flow Reserve) trial assessed the efficacy of a routine post-PCI physiology-guided incremental optimisation strategy (PIOS) in achieving optimal post-PCI FFR results (FFR ≥ 0.90). Patients randomized to the PIOS arm with post-PCI FFR < 0.90 were treated according to a pre-specified protocol based on the findings of the post-PCI FFR pullback (Figure S1). Whilst PIOS improved post-PCI FFR and reduced the proportion of patients with a post-PCI FFR ≤ 0.80, there was no significant difference between groups in the proportion of patients achieving an optimal post-PCI FFR value of ≥0.90 [5]. The pullback pressure gradient (PPG) is a new metric derived from FFR pullbacks that can differentiate focal from diffuse coronary artery disease (CAD) [8]. Although CAD patterns impact post-PCI physiology, their influence of PCI optimisation is unknown. Thus, we sought to investigate the impact of baseline CAD patterns, defined by the PPG, on physiology-guided PCI optimisation in the TARGET-FFR trial.

## 2. Materials and Methods

### 2.1. Study Population

This study is a post hoc analysis of the TARGET-FFR study. Briefly, TARGET-FFR was a prospective, single-centre, randomized, controlled, parallel-group, blinded clinical trial conducted at the Golden Jubilee National Hospital in Glasgow, UK [5]. The study is registered at ClinicalTrials.gov identifier NCT03259815. Patients undergoing PCI for either stable angina, medically stabilised non-ST-segment elevation myocardial infarction (NSTEMI) or staged completion of non-culprit vessel revascularization following either NSTEMI or ST-segment elevation myocardial infarction (STEMI) were eligible for inclusion. A list of the inclusion and exclusion criteria is provided in Table S1. All patients signed informed consent before any study procedure. Patients were randomized to either PIOS or control group [5]. For the present analysis, patients with both pre-PCI FFR pullbacks and post-PCI FFR were considered for inclusion. Coronary physiology data were analysed by a core laboratory (CoreAalst BV, Aalst, Belgium).

### 2.2. Procedures

The details of the coronary physiology measurements and PCI have been described previously [5]. FFR measurements were performed using the PressureWire X Guidewire (Abbott Laboratories, Abbott Park, IL, USA). Following administration of a 200 μg bolus of intracoronary nitrate, the pressure wire sensor was positioned at the tip of the guide catheter and equalised with the aortic pressure. The pressure wire was then advanced to position the sensor in the distal third of the vessel. Hyperaemia was induced by infusion of adenosine into an antecubital vein at a rate of 140 μg/kg/min [9]. Pre-PCI FFR pullbacks were performed manually, and their use and interpretation were left to the operator’s discretion. After angiographically successful PCI, patients were randomized 1:1 to either the PIOS or control groups. In patients randomized to the PIOS group with post-PCI FFR < 0.90, operators planned additional interventions based on the findings of the post-PCI FFR pullback according to the incremental optimisation protocol (Figure S1). Following these additional optimisation measures, FFR was re-assessed, and the procedure was completed. In the control group, post-PCI FFR and pullback information were acquired but concealed from the operator. The use of adjunctive intravascular imaging was left to the operator’s discretion.

### 2.3. Characterisation of CAD Patterns

The PPG index was calculated off-line from the manual pre-PCI FFR pullbacks. Details of the PPG calculation have been described in detail elsewhere [8]. In brief, the PPG combines two parameters extracted from FFR pullback curves: (1) the maximal pressure gradient over 20% of the pullback duration and (2) the length of functional disease computed using a FFR threshold per unit of time. PPG values close to 1 represent focal disease, and values close to 0 characterise diffuse CAD. The PPG was calculated using a commercially available console (Coroflow v3.5, Coroventis Research AP, Uppsala, Sweden). Pressure tracings with ventricularisation, absence of a dicrotic notch, drift of more than 0.05 FFR units, unstable hyperaemic conditions during the pullback manoeuvre, pullback duration less than 15 s, and pullback curves with major artifacts were excluded. Delta FFR was normalised by pre-PCI FFR ((final post-PCI FFR minus pre-PCI FFR divided by one minus pre-PCI FFR) by a factor of one hundred). Post-PCI FFR pullbacks were also quantitatively analysed to determine the magnitude of residual focal pressure gradients. The residual PPG was defined as the maximal pressure gradient, in absolute FFR units, over 20% of the duration of the pullback curve (i.e., the first component of the PPG equation). To assess the outcomes of PIOS stratified by CAD patterns, the population was divided into tertiles according to the baseline PPG distribution, and the highest tertile was considered to be focal disease, whereas the intermediate and low tertiles, comprising patients with both mixed (focal and diffuse) and diffuse CAD, were considered to be diffuse disease [10]. The objective of the present analysis was to investigate the effect of PPG-defined focal vs. diffuse disease on the efficacy of PIOS in improving post-PCI FFR.

### 2.4. Statistical Analysis

Data are expressed as mean ± SD and median (interquartile range) for normally and non-normally distributed data, respectively. Categorical variables are expressed as frequencies and percentages (%). Continuous variables were compared using Student’s *t*-test (or Mann–Whitney tests as appropriate), and categorical variables were compared using the Chi-square test (or Fisher’s exact test as appropriate). Differences across the groups were compared with the Kruskal–Wallis H test. Predictors of the post-PCI FFR value were assessed using univariate and multivariate regression analyses. The variables included in the multivariate analysis were selected based on their known association with final post-PCI FFR [11,12]. Formal interaction testing was performed between the randomization arm (PIOS or controls) and the PPG for the outcome of post-PCI FFR. Receiver operating characteristic (ROC) curve analyses were used to assess the capacity of the residual PPG for predicting final post-PCI FFR ≥ 0.90. All analyses were performed in the intention-to-treat population in patients with pre-PCI FFR pullbacks suitable for PPG calculation. A two-sided *p*-value of 0.05 or less was considered statistically significant. All statistical analyses were performed with R statistical software (R Foundation for Statistical Computing, Vienna, Austria).

## 3. Results

### 3.1. Study Population

Between February 2018 and November 2019, 260 patients were randomized. Of these, 192 patients had pre-PCI FFR pullbacks (PIOS: *n* = 98; control: *n* = 94). Finally, 114 patients (61 patients in the PIOS group and 53 patients in the control group) with pullbacks of sufficient quality for PPG calculation were included in the analysis. The study flowchart is shown in Figure S2.

### 3.2. Baseline Characteristics

Baseline clinical, procedural, and functional characteristics stratified by the randomization arms are shown in Table 1. The mean age was 59.8 ± 8.1 years, 85.1% were male, and the LAD was the most frequently treated vessel in 63.2%. There were no significant differences in clinical, procedural, or functional baseline characteristics between PIOS and controls. Overall, the mean FFR increased after PCI from 0.62 ± 0.14 to 0.85 ± 0.07 (*p* < 0.001). There was no difference in the final post-PCI FFR between PIOS and controls (0.86 ± 0.08 vs. 0.85 ± 0.07; *p*-value = 0.27).

### 3.3. Baseline CAD Patterns and Final Post-PCI FFR

**Figure 1:**
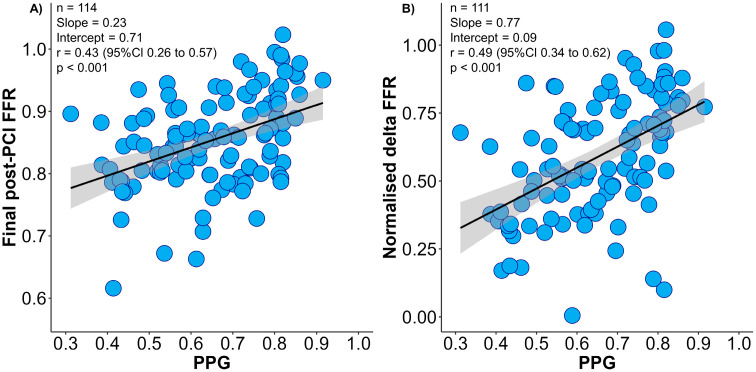
Correlation between pull-back pressure gradient (PPG) and final post-PCI fractional frow reserve (FFR) and normalised delta FFR. Panel (**A**) shows the relationship between the PPG (x-axis) and final post-PCI FFR (y-axis). Panel (**B**) shows the relationship between the PPG (x-axis) and normalised delta FFR (y-axis). PPG had significant correlations with both final post-PCI FFR and normalised delta FFR. Delta FFR was normalised by pre-PCI FFR (post-PCI FFR minus pre-PCI FFR divided by 1 − pre-PCI FFR). FFR = Fractional flow reserve; PCI = percutaneous coronary intervention; PPG = pullback pressure gradient.

The mean PPG value was 0.65 ± 0.14, and focal disease was defined as PPG ≥ 0.74 (highest tertile). The PPG was moderately correlated with normalised delta FFR and final post-PCI FFR (Figure 1). Patients with focal disease achieved significantly larger changes in FFR after PCI leading to higher final post-PCI FFR than patients with diffuse CAD (normalised delta FFR 72.0 ± 20.3% in focal vs. 52.5 ± 19.2% in diffuse, *p* < 0.001). In addition, patients with focal CAD achieved higher post-PCI CFR (Table S2). The proportion of patients achieving optimal final post-PCI FFR ≥ 0.90 was significantly higher in focal disease (52.6% vs. 15.8%; *p*-value < 0.001). In the multivariate analysis, pre-PCI FFR and the PPG were independently associated with final post-PCI FFR (Table S3).

### 3.4. Stent Optimisation in Focal and Diffuse Disease

**Figure 2:**
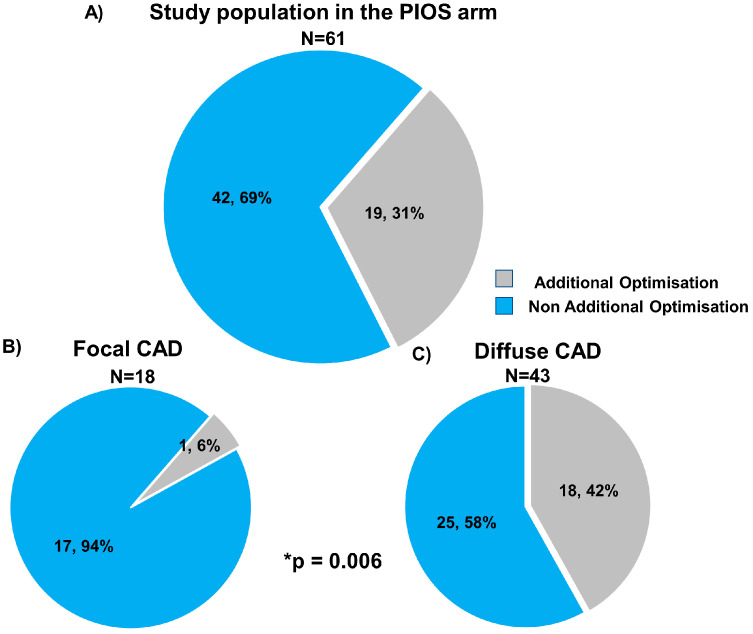
Use of physiology-guided optimisation stratified by focal and diffuse coronary artery disease. Panel (**A**) shows the frequency of additional optimisation in overall population in the PIOS arm (grey colour). Panel (**B**) shows the frequency of additional optimisation in focal CAD, and panel (**C**) shows the frequency of additional optimisation in diffuse CAD. CAD = Coronary artery disease; PIOS = physiology-guided incremental optimisation strategy. * Comparison frequency of additional optimisation between focal and diffuse CAD. Categorical variables are expressed as number and percentage *n*, %.

**Figure 3:**
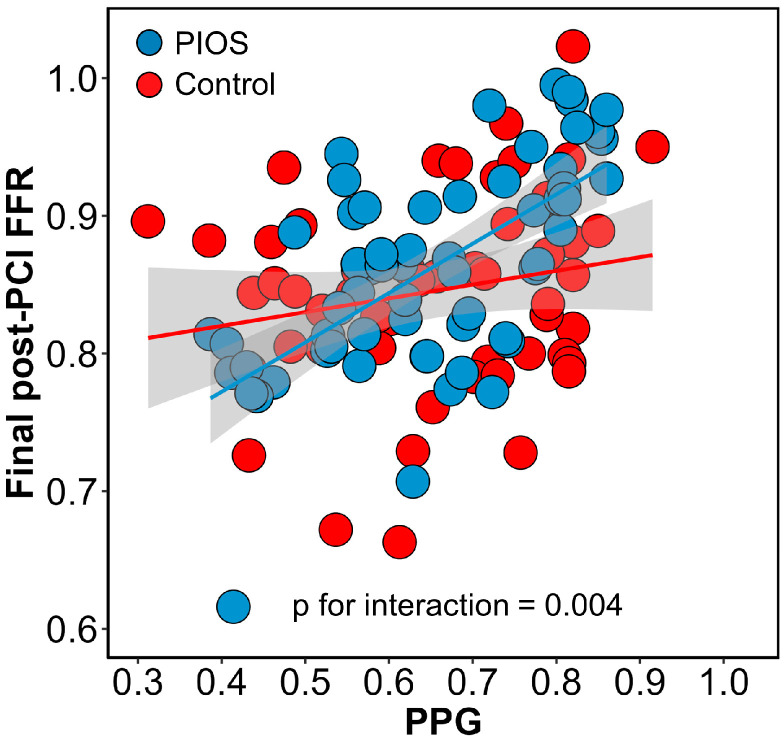
Interaction between pullback pressure gradient (PPG) and randomization arms. Interaction between PPG and randomization arms for final post-PCI FFR. The blue dots and line represent patients in the PIOS arm, and the red dots and line represent the control arm. There was a significant interaction between randomization groups. FFR = Fractional flow reserve; PCI = percutaneous coronary intervention; PIOS = physiology-guided incremental optimisation strategy; PPG = pullback pressure gradient.

Post-PCI physiological results stratified by CAD patterns and the randomisation arm are shown in Table 2. Immediately after stenting, patients with focal CAD had higher post-PCI FFR compared to diffuse disease (0.93 ± 0.05 focal PIOS vs. 0.83 ± 0.07 diffuse PIOS vs. 0.87 ± 0.07 focal controls vs. 0.83 ± 0.07 diffuse controls). In the PIOS arm, optimisation was applied more frequently to vessels with diffuse CAD (6% (1/18) vs. 42% (18/43); *p*-value = 0.006). In the 18 patients with diffuse CAD in the PIOS group who received additional optimisation, PIOS, FFR improved from 0.76 ± 0.09 to 0.83 ± 0.05 (*p*-value < 0.01; Figure 2 and Table S4). However, when comparing post-PCI FFR values in patients with diffuse disease, PIOS did not result in higher post-PCI FFR compared to controls (0.83 ± 0.07 PIOS vs. 0.83 ± 0.07 control; *p*-value = 0.90, Figure S3). In contrast, patients with focal CAD achieved higher post-PCI FFR, and those with focal CAD randomized to PIOS achieved significantly higher final post-PCI FFR (0.93 ± 0.05 focal PIOS vs. 0.83 ± 0.07 diffuse PIOS vs. 0.87 ± 0.07 focal controls vs. 0.83 ± 0.07 diffuse controls; *p*-value < 0.001; Figure S3). Moreover, there was a significant interaction between PPG and the randomization arm for post-PCI FFR (*p*-value for interaction = 0.004; Figure 3). The proportion of patients achieving post-PCI FFR ≥ 0.90 stratified by CAD patterns and the randomization arm is shown in Figure S4.

### 3.5. Residual PPG and Final Post-PCI FFR

**Figure 4:**
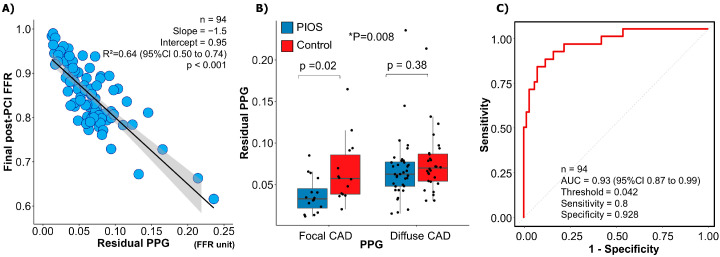
Residual pullback pressure gradient (PPG) and post-PCI fractional flow reserve (FFR). Panel (**A**) shows the relationship between the residual PPG (x-axis) and final post-PCI FFR (y-axis). Residual PPG had a significant correlation with final post-PCI FFR. Panel (**B**) demonstrates differences in residual FFR between the focal PIOS, diffuse PIOS, focal control, and diffuse control groups. * Focal CAD PIOS vs. diffuse CAD PIOS vs. focal CAD controls vs. diffuse CAD controls. Panel (**C**) shows ROC curve analysis for the capacity of residual PPG for predicting final post-PCI FFR ≥ 0.90. AUC = Area under curve; CAD = coronary artery disease; FFR = fractional flow reserve; PIOS = physiology-guided incremental optimisation strategy; PPG = pullback pressure gradient; ROC = receiver operating characteristic.

The mean residual diameter stenosis was 15.0 ± 8.8%, and there was no difference between focal and diffuse CAD (16.0 ± 9.9% focal vs. 14.5 ± 8.2% diffuse; *p*-value = 0.41) or between PIOS and controls (14.5 ± 9.2% PIOS vs. 15.6 ± 8.4% control; *p*-value = 0.50). The mean residual PPG derived from post-PCI FFR pullbacks was 0.07 ± 0.04. The residual PPG conditioned final post-PCI FFR (R^2^ = 0.64, 95% CI 0.50 to 0.74, *p* < 0.001; Figure 4A). The residual PPG was not significantly different between patients randomized to PIOS versus controls (0.06 ± 0.04 PIOS vs. 0.07 ± 0.04 controls; *p*-value = 0.06). However, patients with focal CAD randomized to PIOS had the lowest residual PPG (0.04 ± 0.02 focal PIOS vs. 0.07 ± 0.04 focal control, 0.07 ± 0.04 diffuse PIOS vs. 0.08 ± 0.04 diffuse control; ANOVA *p*-value = 0.008; Figure 4B). The residual PPG predicted final post-PCI FFR ≥ 0.90 with an AUC of 0.93 (95% CI: 0.87–0.99; *p* < 0.001; Figure 4C).

### 3.6. Clinical Outcomes

At a median follow-up of 2 years, only one target vessel failure (TVF) occurred. This patient, randomized to PIOS with focal disease, suffered a presumed cardiac death 17 months after the procedure. There were no differences in TVF between PIOS and controls or between patients with focal or diffuse CAD.

## 4. Discussion

The main findings of the present study can be summarized as follows: (1) pathophysiological CAD patterns, defined by the PPG index, correlated with post-PCI FFR and delta FFR. Patients with focal disease achieved higher post-PCI FFR compared to those with diffuse CAD. (2) Physiology-guided PCI optimisation was more frequently applied to patients with diffuse CAD (as the incidence of post-PCI FFR < 0.90 was higher). However, despite the higher use of optimisation, final FFR values in patients with diffuse disease did not differ between PIOS and controls. (3) There was a significant interaction between the PPG and the randomization arm for post-PCI FFR. Patients with focal disease randomized to PIOS achieved the highest post-PCI FFR. (4) Post-PCI FFR was largely determined by focal residual pressure gradients and the residual PPG index, which was significantly lower in patients with focal disease randomized to stent optimisation.

The PPG is the first quantitative metric to differentiate focal from diffuse disease. In line with previous publications, the PPG correlated with post-PCI and delta FFR [13]. Patients with a high PPG (focal disease) achieved significantly higher post-PCI FFR and delta FFR. We demonstrated that improvement in coronary blood flow with PCI partially depends on the baseline pattern of disease. The higher post-PCI FFR in focal disease was observed independent of the application of physiology-guided stent optimisation. In contrast, in diffuse disease, additional optimisation had limited value in terms of post-PCI FFR, and, in this group, the final FFR was similar between patients randomized to PIOS and controls. The advent of the PPG, a reproducible metric for quantifying the physiological pattern of CAD, will facilitate the study of treatment options stratified by disease patterns.

TARGET-FFR was the first randomized trial assessing the feasibility of routine post-PCI FFR-guided optimisation strategy [14]. As shown in this study, physiologic outcomes after PCI are influenced by the baseline CAD pattern. By design, TARGET-FFR applied physiology-guided stent optimisation to patients with predominantly diffuse disease because these additional manoeuvres were triggered by low post-PCI FFR, a proxy of diffuse disease. In addition, the high proportion of vessels with diffuse CAD in the study limited the efficacy of PIOS in improving the post-PCI FFR. Similarly, the mechanism leading to higher post-PCI FFR in patients with focal CAD randomized to PIOS is likely unrelated to the application of optimisation manoeuvres since only one patient with focal CAD in the PIOS group received an additional intervention. Nonetheless, it can be hypothesised that patients with focal disease pre-PCI and a suboptimal functional result after stenting might have additional targets for optimisation (e.g., unmasked focal pressure gradients arising from the untreated segments), which may lead to improved post-PCI physiology. The hypothesis that functionally guided PCI optimisation is more effective in focal vs. diffuse disease requires further investigation.

There are mainly three mechanisms leading to unsatisfactory PCI results: first, the presence of residual pressure gradients within the stented segment, mainly associated with stent underexpansion or issues at the stent edges [15,16]; second, focal pressure gradients distal or proximal to the treated segment; and third, diffuse residual disease. The first two can potentially be addressed by additional post-dilation or PCI with a resultant improvement in intracoronary haemodynamics. In this study, half of the cases were optimised with additional stent post-dilation, and half underwent an additional PCI. Nonetheless, in the setting of diffuse disease optimisation manoeuvres will result in minor coronary physiology improvement as shown in this cohort by the small increase in FFR from 0.80 ± 0.09 immediately after stenting to 0.83 ± 0.07 after optimisation. In this work, we also introduced the residual PPG as a metric to quantify residual pressure gradients. Distinctively from the original study protocol, in which residual focal pressure gradients were assessed visually, the residual PPG provides an automatic quantification of residual focal gradients after stenting. This new approach leverages the original PPG formula adapted to the post-PCI setting to quantify focal pressure drops in absolute FFR units. As anticipated, the residual PPG was strongly associated with post-PCI FFR with an AUC of 0.93 (95% CI: 0.87–0.99). Interestingly, the residual PPG was significantly lower in patients with focal CAD assigned to PIOS highlighting the influence of the baseline pattern of disease and potentially the value of optimisation in the magnitude of residual focal gradients after PCI. The clinical relevance of focal residual translesional gradients using angiography-derived FFR software in the post-PCI phase has previously been suggested in one study [17]. Nonetheless, further studies are required to better understand the clinical implications of residual PPG better. The importance of this study is identifying which patients benefit from additional physiology-guided optimisation. Patients randomized to PIOS with focal CAD patterns achieved greater improvement in post-PCI FFR compared to patients with diffuse CAD. Therefore, using CAD patterns stratified by PPG before stenting identifies patients who are likely to achieve high post-PCI FFR.

This study has several limitations. First, pre-PCI FFR pullbacks were only performed in 74% (192/260) of cases, of which 59.3% (114/192) were deemed suitable for PPG calculation. However, this attrition of the sample size was equally distributed between the two arms, and the benefit of randomisation in terms of baseline risk distribution was preserved. Second, the study was not powered to assess clinical outcomes; the primary objective was post-PCI FFR, a surrogate endpoint of adverse events after PCI. Third, the PPG and residual PPG were calculated off-line, limiting the evaluation of the clinical utility of these metrics. Nonetheless, the quantification of pullback data has the potential to standardise diagnosis and treatment pathways, accounting for CAD patterns and residual disease. Further studies are required to better understand the pattern of CAD via non-invasive assessment using coronary blood flow models [18,19,20]. Finally, this is a post hoc analysis of randomized clinical trials, and all the findings presented in this study should be interpreted as hypothesis generating.

## 5. Conclusions

Baseline coronary artery disease patterns influence FFR after PCI. Patients with diffuse disease (low PPG) achieve lower post-PCI FFR compared to those with focal CAD. Physiology-guided stent optimisation was applied more frequently to vessels with diffuse disease; however, despite these patients with focal CAD at baseline achieved higher post-PCI FFR. Characterising coronary artery disease patterns with the PPG before stenting identifies patients likely to achieve high post-PCI FFR.

## Figures and Tables

**Table 1:** Baseline characteristics.

Variables	All	PIOS	Control	*p*-Value
*N*	114	61	53	
Clinical characteristics				
Gender (male), *n* (%)	97 (85.1)	53 (86.9)	44 (83.0)	0.75
Age (years), mean ± SD	59.8 ± 8.1	59.3 ± 8.0	60.4 ± 8.2	0.45
BMI, mean ± SD	29.7 ± 4.7	29.4 ± 4.3	30.1 ± 5.2	0.46
Family history, *n* (%)	75 (65.8)	40 (65.6)	35 (66.0)	1.00
Smoking, *n* (%)	80 (70.2)	44 (72.1)	36 (67.9)	0.78
Hypertension, *n* (%)	50 (43.9)	29 (47.5)	21 (39.6)	0.51
Dyslipidaemia, *n* (%)	65 (57.0)	33 (54.1)	32 (60.4)	0.63
Diabetes mellitus, *n* (%)	23 (20.2)	10 (16.4)	13 (24.5)	0.40
History of stroke, *n* (%)	7 (6.1)	5 (8.2)	2 (3.8)	0.56
Chronic kidney disease, *n* (%)	3 (2.6)	2 (3.3)	1 (1.9)	1.00
Prior PCI, *n* (%)	51 (44.7)	31 (50.8)	20 (37.7)	0.23
Prior MI, *n* (%)	50 (43.9)	29 (47.5)	21 (39.6)	0.51
Prior CABG, *n* (%)	0	0	0	NA
Symptomatic angina, *n* (%)	95 (69.2)	53 (86.9)	42 (79.2)	0.95
CCS I	21 (22.1)	12 (22.6)	9 (21.4)	
CCS II	48 (50.5)	26 (49.1)	22 (52.4)	
CCS III	26 (27.4)	15 (28.3)	11 (26.2)	
CCS IV	0 (0)	0 (0)	0 (0)	
Clinical presentation, *n* (%)				0.22
Stable angina	29 (25.4)	13 (21.3)	16 (30.2)	
ACS—NSTEMI	44 (38.6)	21 (34.4)	23 (43.4)	
ACS—Unstable angina	1 (0.9)	1 (1.6)	0 (0.0)	
Staged PCI	40 (35.1)	26 (42.6)	14 (26.4)	
Procedural characteristics				
Vessel, *n* (%)				0.99
LAD	72 (63.2)	38 (62.3)	34 (64.2)	
Non-LAD	42 (36.8)	23 (37.7)	19 (35.8)	
QCA diameter stenosis (%), mean	61.0	60.9	61.2	0.92
QCA lesion length (mm), median (IQR)	10.8 [7.96, 13.2]	10.7 [8.34, 13.6]	10.9 [6.93, 13.0]	0.73
Intravascular imaging, *n* (%)	22 (19.3)	10 (16.4)	12 (22.6)	0.55
IVUS, *n* (%)	19 (86.4)	10 (100)	9 (77.4)	
OCT, *n* (%)	3 (13.6)	0 (0.0)	3 (23.1)	
Stent diameter (mm), median (IQR)	3.00 [3.0, 3.5]	3.00 [3.0, 3.5]	3.00 [3.0, 3.5]	0.45
Stent length (mm), median (IQR)	32.0 [23.0, 38.0]	32.0 [23.0, 38.0]	28.0 [23.0, 38.0]	0.76
Number of stents per patients, median (IQR)	1.00 [1.00, 2.00]	1.00 [1.00, 2.00]	1.00 [1.00, 2.00]	0.97
Total stent length (mm), median (IQR)	38.0 [25.0, 50.8]	33.0 [28.0, 52.0]	38.0 [24.0, 50.0]	0.76
Physiological characteristics				
Pre-PCI Pd/Pa*, median (IQR)	0.85 [0.75, 0.90]	0.86 [0.81, 0.91]	0.83 [0.72, 0.89]	0.11
Pre-PCI CFR*, median (IQR)	2.03 [1.47, 2.61]	2.11 [1.49, 2.60]	1.99 [1.31, 2.60]	0.43
Pre-PCI IMR*, median (IQR)	22.9 [16.6, 31.8]	24.0 [18.0, 33.5]	21.7 [16.0, 31.3]	0.31
Pre-PCI FFR*, median (IQR)	0.62 ± 0.14	0.65 ± 0.12	0.58 ± 0.15	0.04
Final post-PCI FFR*, mean ± SD	0.85 ± 0.07	0.86 ± 0.08	0.85 ± 0.07	0.27
Normalised delta FFR*	59.2 ± 21.6	59.4 ± 20.8	58.9 ± 22.7	0.91
Final post-PCI FFR ≤ 0.80 (%), *n* (%)	27 (23.7)	13 (21.3)	14 (26.4)	0.68
Final post-PCI FFR ≥ 0.80 (%), *n* (%)	88 (77.2)	48 (78.7)	40 (75.5)	0.85
Final post-PCI FFR ≥ 0.90 (%), *n* (%)	32 (28.1)	22 (36.1)	10 (18.9)	0.07
PPG, median (IQR)	0.66 [0.55, 0.78]	0.64 [0.56, 0.79]	0.68 [0.54, 0.79]	0.65
Residual PPG*	0.07 ± 0.04	0.06 ± 0.04	0.07 ± 0.04	0.06

Categorical variables are expressed as number and percentage. Continuous variables are indicated as median (interquartile range). BMI, Body mass index; CABG, coronary artery bypass graft; CCS, Canadian cardiovascular society; CFR, coronary flow reserve; FFR, fractional flow reserve; IMR, index of microvascular resistance; IVUS, intravascular ultrasound; LAD, left anterior descending artery; LCX, left circumflex artery; Pa, aortic pressure; PCI, percutaneous coronary intervention; Pd, distal coronary pressure; PDA, posterior descending artery; PLA, posterior lateral artery; PPG, pullback pressure gradient; MI, myocardial infarction; NA, not applicable; NSTE-ACS, non-ST elevation acute coronary syndrome; RCA, right coronary artery; QCA, quantitative coronary angiography; OCT, optical coherence tomography. Normalised delta FFR was normalised by pre-PCI FFR ((final post-PCI FFR minus pre-PCI FFR divided by one minus pre-PCI FFR) by a factor of one hundred). Pre-PCI Pd/Pa*: *N* = 110 (PIOS = 60; control = 50); pre-PCI CFR*: *N* = 107 (PIOS = 58; control = 49); pre-PCI IMR*: *N* = 104 (PIOS = 56; control = 48); pre-PCI FFR*: *N* = 111 (PIOS = 61; control = 50); final post-PCI FFR*: *N* = 114 (PIOS 61; control 53); normalised delta FFR*: *N* = 111 (PIOS = 61; control = 50); residual PPG*: *N* = 94 (PIOS = 52; control = 42).

**Table 2:** Comparison of functional characteristics between randomized groups stratified by physiological pattern of CAD.

Variables	PIOS			Controls			*p*-Value ***
	Focal CAD	Diffuse CAD	*p*-Value *	Focal CAD	Diffuse CAD	*p*-Value **	
Number, (%)	18 (29.5)	43 (70.5)		20 (37.7)	33 (62.3)		
Vessel (%)			<0.001			<0.001	<0.001
LAD, *n* (%)	2 (11.1)	36 (83.7)		6 (30.0)	28 (84.8)		
LCx, *n* (%)	10 (55.6)	2 (4.7)		3 (15.0)	2 (6.1)		
RCA, *n* (%)	7 (33.3)	3 (11.6)		11 (55.0)	3 (9.1)		
Baseline coronary physiology							
Pd/Pa, median (IQR)	0.93 [0.83, 0.95]	0.85 [0.81, 0.88]	0.01	0.76 [0.69, 0.91]	0.85 [0.76, 0.89]	0.77	0.048
CFR, median (IQR)	2.00 [1.78, 2.21]	2.25 [1.42, 2.69]	0.33	1.71 [1.21, 2.012	2.24 [1.66 2.71]	0.07	0.19
IMR, median (IQR)	25.1 [19.9, 37.5]	23.9 [17.8, 32.0]	0.55	24.1 [18.6, 31.8]	20.8 [15.8, 28.5]	0.42	0.59
FFR, mean ± SD	0.69 ± 0.13	0.63 ± 0.11	0.048	0.55 ± 0.15	0.61 ± 0.15	0.17	0.02
PPG, median (IQR)	0.81 [0.78, 0.82]	0.58 [0.53, 0.66]	<0.001	0.81 [0.78, 0.82]	0.59 [0.48, 0.66]	<0.001	<0.001
Immediately after stenting							
Stent post-dilatation, *n* (%)	18 (100.0)	43 (100.0)	1	18 (90.5)	33 (100.0)	0.27	0.02
Intravascular imaging, *n* (%)	1 (5.6)	9 (20.9)	0.26	0 (0)	12 (36.4)	0.006	0.004
Pd/Pa, median (IQR)	0.98 [0.97, 1.01]	0.90 [0.88, 0.92]	<0.001	0.98 [0.93, 1.00]	0.92 [0.89, 0.94]	0.001	<0.001
CFR, median (IQR)	4.44 [2.39, 5.81]	2.71 [1.97, 4.00]	0.01	3.28 [2.44, 5.41]	2.74 [2.41, 4.13]	0.25	0.04
IMR, median (IQR)	16.1 [13.2, 23.1]	18.9 [13.1, 26.3]	0.31	13.1 [10.7, 19.5]	17.3 [13.1, 22.7]	0.06	0.07
FFR, mean ± SD	0.93 ± 0.05	0.80 ± 0.09	<0.001	0.87 ± 0.07	0.83 ± 0.07	0.09	<0.001
1st PIOS treatments							
1st PIOS treatments performed, *n* (%)	1 (5.6)	18 (41.9)	0.006	-	-		-
Additional stent post-dilatation, *n* (%)	1 (5.6)	11 (25.6)	0.09	-	-		-
Additional lesion treated PCI, *n* (%)	0 (0)	8 (18.6)	0.09	-	-		-
Additional intravascular imaging, *n* (%)	0 (0.0)	1 (2.3)	1	-	-		-
Pd/Pa, median (IQR)	0.98 [0.97, 1.01]	0.90 [0.89, 0.93]	<0.001				-
CFR, median (IQR)	4.44 [2.59, 5.81]	3.46 [2.01, 4.14]	0.07	-	-		-
IMR, median (IQR)	16.1 [13.2, 18.7]	17.1 [13.1, 25.2]	0.52				-
FFR, mean ± SD	0.93 ± 0.05	0.83 ± 0.07	<0.001	-	-		-
2nd PIOS treatments							
2nd PIOS treatments performed, *n* (%)	0 (0)	2 (4.7)	1	-	-		-
Additional stent post-dilatation, *n* (%)	0 (0)	2 (4.7)	1	-	-		-
Additional lesion treated, *n* (%)	0 (0)	2 (4.7)	1	-	-		-
Additional intravascular imaging, *n* (%)	0 (0)	2 (4.7)	1	-	-		-
Pd/Pa, median (IQR)	0.98 [0.97, 1.01]	0.91 [0.90, 0.93]	<0.001	-	-		-
CFR, median (IQR)	4.44 [2.59, 5.81]	3.23 [2.01, 4.11]	0.051	-	-		-
IMR, median (IQR)	16.1 [13.2, 18.7]	17.6 [13.1, 25.2]	0.49	-	-		-
FFR, mean ± SD	0.93 ± 0.05	0.83 ± 0.07	<0.001	-	-		-
Final coronary physiology							
Number of stents per patients, median (IQR)	1.00 [1.00, 1.00]	1.00 [1.00, 2.00]	0.14	1.00 [1.00, 2.00]	1.00 [1.00, 2.00]	0.28	0.34
Total stent length (mm), median (IQR)	32.0 [25.0, 36.8]	38.0 [28.0, 56.0]	0.12	38.0 [30.3, 48.0]	38.0 [24.0, 56.0]	0.93	0.47
Pd/Pa, median (IQR)	0.98 [0.97, 1.01]	0.91 [0.90, 0.93]	<0.001	0.98 [0.93, 1.00]	0.92 [0.89, 0.94]	0.001	<0.001
CFR, median (IQR)	4.44 [2.59, 6.14]	3.23 [2.01, 4.11]	0.051	3.28 [2.44, 5.41]	2.74 [2.41, 4.13]	0.25	0.18
IMR, median (IQR)	16.1 [13.2, 18.7]	17.6 [13.01 25.2]	0.53	13.1 [10.7, 19.5]	17.3 [13.1, 22.7]	0.07	0.17
FFR, mean ± SD	0.93 ± 0.05	0.83 ± 0.07	<0.001	0.87 ± 0.07	0.83 ± 0.07	0.057	<0.001
Delta Pd/Pa, median (IQR)	0.06 [0.04, 0.15]	0.06 [0.03, 0.10]	0.82	0.20 [0.07, 0.28]	0.06 [0.04, 0.19]	0.079	0.09
Delta CFR, median (IQR)	2.36 [0.79, 3.18]	0.63 [−0.05, 1.92]	0.01	1.60 [0.78, 2.90]	1.04 [0.20, 1.97]	0.11	0.03
Delta IMR, median (IQR)	−8.23 [−13.2, −3.09]	−3.55 [−9.98, −0.28]	0.19	−11.3 [−15.1, −5.67]	−3.13 [−9.71, 1.26]	0.04	0.10
Normalised delta FFR (%), mean ± SD	76.5 ± 15.5	52.2 ± 18.5	<0.001	67.8 ± 23.5	53.0 ± 20.5	0.02	<0.001
FFR ≥ 0.90 (%), *n* (%)	14 (77.8)	8 (18.6)	<0.001	6 (30.0)	4 (12.1)	0.21	<0.001
FFR ≥ 0.80 (%), *n* (%)	0 (0.0)	13 (30.2)	0.02	5 (25.0)	9 (27.3)	0.79	0.07
Residual PPG	0.04 ± 0.02	0.07 ± 0.04	0.004	0.07 ± 0.04	0.08 ± 0.04	0.44	0.008

Categorical variables are expressed as number and percentage. Continuous variables are indicated as median (interquartile range). CFR, Coronary flow reserve; FFR, fractional flow reserve; IMR, index of microvascular resistance; Pa, aortic pressure; Pd, distal coronary pressure; PPG, pullback pressure gradient. Normalised delta FFR was normalised by pre-PCI FFR ((final post-PCI FFR minus pre-PCI FFR divided by one minus pre-PCI FFR) by a factor of one hundred. Pre-PCI Pd/Pa*: *N* = 110 (PIOS/Focal = 18, PIOS/Diffuse = 42, Control/Focal = 20, Control/Diffuse = 30); pre-PCI CFR: *N* = 107 (PIOS/Focal = 17, PIOS/Diffuse = 41, Control/Focal = 18, Control/Diffuse = 31); pre-PCI IMR: *N* = 104 (PIOS/Focal = 18, PIOS/Diffuse = 38, Control/Focal = 16, Control/Diffuse = 32); pre-PCI FFR: *N* = 111 (PIOS/Focal = 18, PIOS/Diffuse = 43, Control/Focal = 20, Control/Diffuse = 30); immediately after stenting Pd/Pa: *N* = 113 (PIOS/Focal = 18, PIOS/Diffuse = 42, Control/Focal = 20, Control/Diffuse = 33); immediately after stenting CFR: *N* = 112 (PIOS/Focal = 18, PIOS/Diffuse = 43, Control/Focal = 20, Control/Diffuse = 31); immediately after stenting IMR: *N* = 112 (PIOS/Focal = 18, PIOS/Diffuse = 43, Control/Focal = 20, Control/Diffuse = 31); immediately after stenting FFR: *N* = 113 (PIOS/Focal = 18, PIOS/Diffuse = 42, Control/Focal = 20, Control/Diffuse = 33); after 1st PIOS Pd/Pa: *N* = 114 (PIOS/Focal = 18, PIOS/Diffuse = 43, Control/Focal = 20, Control/Diffuse = 33); after 1st PIOS CFR: *N* = 109 (PIOS/Focal = 18, PIOS/Diffuse = 40, Control/Focal = 20, Control/Diffuse = 31); after 1st PIOS IMR: *N* = 109 (PIOS/Focal = 18, PIOS/Diffuse = 40, Control/Focal = 20, Control/Diffuse = 31); after 1st PIOS FFR: *N* = 114 (PIOS/Focal = 18, PIOS/Diffuse = 43, Control/Focal = 20, Control/Diffuse = 33); after 2nd PIOS Pd/Pa: *N* = 114 (PIOS/Focal = 18, PIOS/Diffuse = 43, Control/Focal = 20, Control/Diffuse = 33); after 2nd PIOS CFR: *N* = 109 (PIOS/Focal = 18, PIOS/Diffuse = 40, Control/Focal = 20, Control/Diffuse = 31); after 2nd PIOS IMR: *N* = 109 (PIOS/Focal = 18, PIOS/Diffuse = 40, Control/Focal = 20, Control/Diffuse = 31); after 2nd PIOS FFR: *N* = 114 (PIOS/Focal = 18, PIOS/Diffuse = 43, Control/Focal = 20, Control/Diffuse = 33); final post-PCI Pd/Pa: *N* = 114 (PIOS/Focal = 18, PIOS/Diffuse = 43, Control/Focal = 20, Control/Diffuse = 33); final post-PCI CFR: *N* = 109 (PIOS/Focal = 18, PIOS/Diffuse = 40, Control/Focal = 20, Control/Diffuse = 31); final post-PCI IMR: *N* = 109 (PIOS/Focal = 18, PIOS/Diffuse = 40, Control/Focal = 20, Control/Diffuse = 31); final post-PCI FFR: *N* = 114 (PIOS/Focal = 18, PIOS/Diffuse = 43, Control/Focal = 20, Control/Diffuse = 33); delta PdPa: *N* = 110 (PIOS/Focal = 18, PIOS/Diffuse = 42, Control/Focal = 20, Control/Diffuse = 30); delta CFR: *N* = 103 (PIOS/Focal = 17, PIOS/Diffuse = 39, Control/Focal = 18, Control/Diffuse = 29); delta IMR: *N* = 103 (PIOS/Focal = 17, PIOS/Diffuse = 39, Control/Focal = 18, Control/Diffuse = 29); normalised delta FFR: *N* = 111 (PIOS/Focal = 18, PIOS/Diffuse = 43, Control/Focal = 20, Control/Diffuse = 30); residual PPG *N* = 94 (PIOS/Focal = 16, PIOS/Diffuse = 36, Control/Focal = 14, Control/Diffuse = 28). * Focal CAD vs. diffuse CAD among PIOS group. ** Focal CAD vs. diffuse CAD among the control group. *** Focal CAD PIOS vs. diffuse CAD PIOS vs. focal CAD controls vs. diffuse CAD controls.

## Data Availability

The data presented in this study are available upon request from the corresponding author. The data are not publicly available due to patient privacy concerns.

## Supplementary Materials

The following supporting information can be downloaded at https://www.mdpi.com/article/10.3390/diagnostics13152612/s1. Table S1: Inclusion criteria/exclusion criteria in TARGET FFR trial; Table S2: Comparison of functional characteristics between focal and diffuse coronary artery disease (CAD); Table S3: Multivariate regression of post-PCI fractional flow reserve (FFR) with pullback pressure gradient (PPG); Table S4: Comparison of fractional characteristics between focal and diffuse coronary artery disease in patients with additional optimisation; Figure S1: Physiology-guided incremental optimisation strategy; Figure S2: Study flowchart; Figure S3: Final post-PCI fractional flow reserve (FFR) stratified by randomization arm and PPG-defined focal or diffuse disease; Figure S4: The rate of optimal post-PCI fractional flow reserve (FFR) (≥0.90) stratified by randomization arm and coronary artery disease.
