# Supplementary material for: Fractional Flow Reserve-Guided Stent Optimisation in Focal and Diffuse Coronary Artery Disease
Source: Diagnostics (Basel). 2023 Aug 7;13(15):2612. doi: 10.3390/diagnostics13152612 (PMC10417445; doi:10.3390/diagnostics13152612)
Supplement: Supplementary file 1 [file diagnostics-13-02612-s001.zip › diagnostics-2485232-supplementary.pdf]

Supplemental Material

Table of contents

Table S1. Inclusion criteria/exclusion criteria in TARGET FFR trial.

Table S2. Comparison of functional characteristics between focal and diffuse coronary artery disease (CAD).

Table S3. Multivariate regression of post-PCI fractional flow reserve (FFR) with pullback pressure gradient (PPG).

Table S4. Comparison of fractional characteristics between focal and diffuse coronary artery disease in patients with additional optimization.

Figure of contents

Figure S1. Physiology-guided incremental optimization strategy.

Figure S2. Study flowchart

Figure S3. Final post-PCI fractional flow reserve (FFR) stratified by randomization arm and PPG defined focal or diffuse disease.

Figure S4. The rate of optimal post-PCI fractional flow reserve (FFR) (≥0.90) stratified by randomization arm and coronary artery disease.

Table S1. Inclusion criteria/exclusion criteria in the TARGET FFR study

**Inclusion criteria**
Patients >18 years of age with coronary artery disease including stable angina and NSTEMI
Participants must be able to provide informed consent
**Exclusion criteria**
PCI in a coronary artery bypass graft
• PCI to an ISR lesion
• PCI to a target artery providing Rentrop grade 2 or 3 collateral blood supply to another vessel
• Inability to receive adenosine (eg, severe reactive airway disease, marked hypotension, or advanced atrioventricular block without pacemaker).
• Recent (within 1 week prior to cardiac catheterisation) STEMI in any arterial distribution (not specifically target lesion).
Severe cardiomyopathy (LVEF <30%)
• Renal insufficiency such that an additional 20 to 30 mL of contrast would, in the opinion of the operator, pose unwarranted risk to the patient.

ISR = in-stent restenosis; LVEF = left ventricular ejection fraction; PCI = percutaneous coronary intervention; STEMI = ST-elevated myocardial infarction; PPG = pullback pressure gradient.

Table S2. Comparison of functional characteristics between focal and diffuse coronary artery disease (CAD)

Variables	Focal CAD	Diffuse CAD	P
N	**38**	**76**	
Physiological characteristics			
Pre PCI Pd/Pa, median [IQR]	0.87 [0.74, 0.95]	0.85 [0.79, 0.88]	0.21
Pre PCI CFR, median [IQR]	1.87 [1.40, 2.15]	2.25 [1.48, 2.71]	0.052
Pre PCI IMR, median [IQR]	24.5 [19.5, 33.9]	21.7 [16.6, 31.2]	0.36
Pre PCI FFR, median [IQR]	0.61±0.16	0.62±0.13	0.73
Final Post PCI Pd/Pa, median [IQR]	0.98 [0.95, 1.01]	0.91 [0.89, 0.93]	<0.001
Final Post PCI CFR, median [IQR]	3.64 [2.47, 5.51]	3.05 [2.10, 4.13]	0.004
Final Post PCI IMR, median [IQR]	14.4 [11.5, 19.2]	17.3 [13.0, 23.5]	0.052
Final Post PCI FFR, mean ± SD	0.90±0.07	0.83±0.07	<0.001
Normalised delta FFR (%), mean ± SD	72.0 ± 20.3	52.5 ± 19.2	<0.001
Final FFR ≤ 0.80 (%), n (%)	5 (13.2)	22 (28.9)	0.10
Final FFR ≥ 0.80 (%), n (%)	34 (89.5)	54 (71.1)	0.048
Final FFR ≥ 0.90 (%), n (%)	20 (52.6)	12 (15.8)	<0.001
PPG, median [IQR]	0.81 [0.78, 0.82]	0.58 [0.49, 0.66]	<0.001

Categorical variables are expressed as number and percentage. Continuous variables are indicated as median (interquartile range). CFR = coronary flow reserve; FFR = fractional flow reserve; IMR = index of microvascular resistance; Pa = aortic pressure; Pd = distal coronary pressure; PPG = pullback pressure gradient.

Normalised delta FFR was normalised by pre-PCI FFR ([final post-PCI FFR minus pre-PCI FFR divided by one minus pre-PCI FFR] by a factor of one hundred).

Table S3. Multivariate regression analyses of post-PCI FFR with pullback pressure gradient (PPG).

Variables	Multivariate analysis		
	**Estimate**	**CI**	**P value**
Age	-0.001	[-0.002, 0.001]	0.36
Gender (male)	-0.035	[-0.070, 0.000]	0.051
Renal failure	-0.003	[-0.080, 0.074]	0.93
Hypertension	0.005	[-0.020, 0.030]	0.69
Diabetes	-0.018	[-0.049, 0.014]	0.27
Pre-PCI FFR	0.175	[0.084, 0.266]	<0.001
PPG	0.24	[0.153, 0.328]	<0.001
PIOS	0.006	[-0.019, 0.031]	0.63

PCI = percutaneous coronary intervention; FFR = fractional flow reserve; PPG = pullback pressure gradient, PIOS = physiology-guided incremental optimisation strategy.

Table S4. Comparison of fractional characteristics between focal and diffuse CAD in patients with additional optimisation.

Variables	Focal CAD	Diffuse CAD	P
N	**1**	**18**	
Pre PCI			
Pre-PCI Pd/Pa, median [IQR]	0.83 [0.83, 0.83]	0.86 [0.82, 0.88]	0.86
Pre-PCI CFR, median [IQR]	2.00 [2.00, 2.00]	2.35 [1.57, 2.61]	0.63
Pre-PCI IMR, median [IQR]	20.6	26.5±7.39	NA
FFR Pre PCI, median [IQR]	0.61 [0.61, 0.61]	0.64 [0.56, 0.67]	0.72
PPG, mean ± SD	0.74	0.56±0.10	NA
Immediately after stenting			
Pd/Pa, median [IQR]	0.89 [0.89, 0.89]	0.90 [0.88, 0.91]	0.62
CFR, median [IQR]	2.06 [2.06, 2.06]	2.51 [2.05, 3.81]	0.47
IMR, median [IQR]	31.5 [31.5, 31.5]	22.4 [17.8, 27.1]	0.27
FFR, median, mean ± SD	0.80	0.76±0.09	NA
1st PIOS treatments			
Pd/Pa, median [IQR]	0.89 [0.89, 0.89]	0.90 [0.90, 0.92]	0.19
CFR, median [IQR]	3.59 [3.59, 3.59]	3.91 [3.17, 5.58]	0.45
IMR, mean ± SD	17.4	20.0±7.67	NA
FFR, mean ± SD	0.81	0.83±0.05	NA
2nd PIOS treatments			
Pd/Pa, median [IQR]	0.89 [0.89, 0.89]	0.91 [0.90, 0.92]	0.14
CFR, median [IQR]	3.59 [3.59, 3.59]	3.76 [2.69, 4.95]	0.59
IMR, mean ± SD	15.3±2.96	20.2±7.52	NA
FFR, mean ± SD	0.81	0.83±0.05	NA
Final coronary physiology			
Pd/Pa, median [IQR]	0.89 [0.89, 0.89]	0.91 [0.90, 0.92]	0.14
CFR, median [IQR]	3.59 [3.59, 3.59]	3.76 [2.69, 4.95]	0.59
IMR, mean ± SD	17.4	20.0±7.67	NA
FFR, mean ± SD	0.81	0.83±0.05	NA

Categorical variables are expressed as number and percentage. Continuous variables are indicated as median (interquartile range). CAD = coronary artery disease; CFR = coronary flow reserve; FFR = fractional flow reserve; IMR = index of microvascular resistance; Pa = aortic pressure; Pd = distal coronary pressure; PPG = pullback pressure gradient.

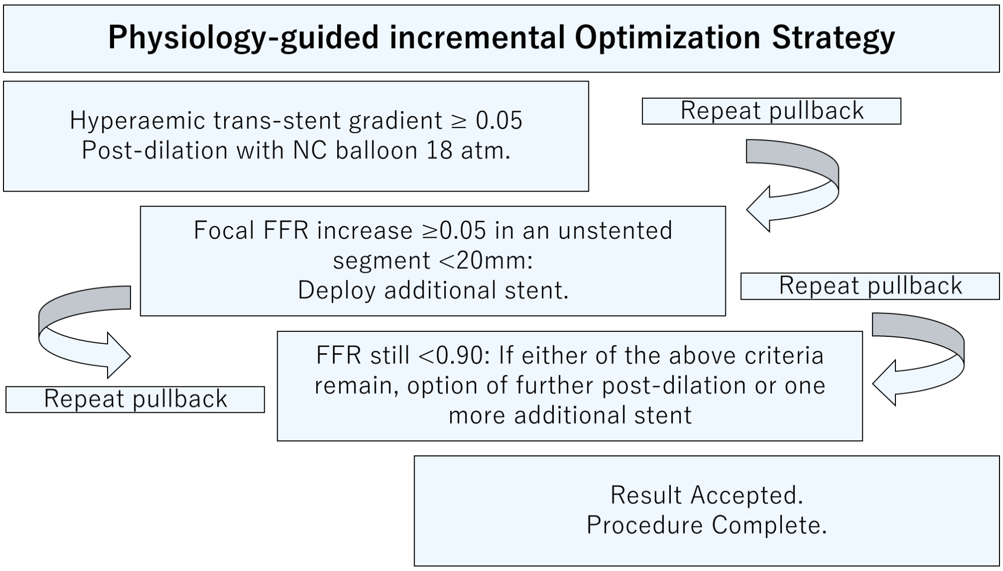

Figure S1. Physiology-guided incremental optimization strategy. FFR = fractional flow reserve; NC balloon = non-complainant balloon.

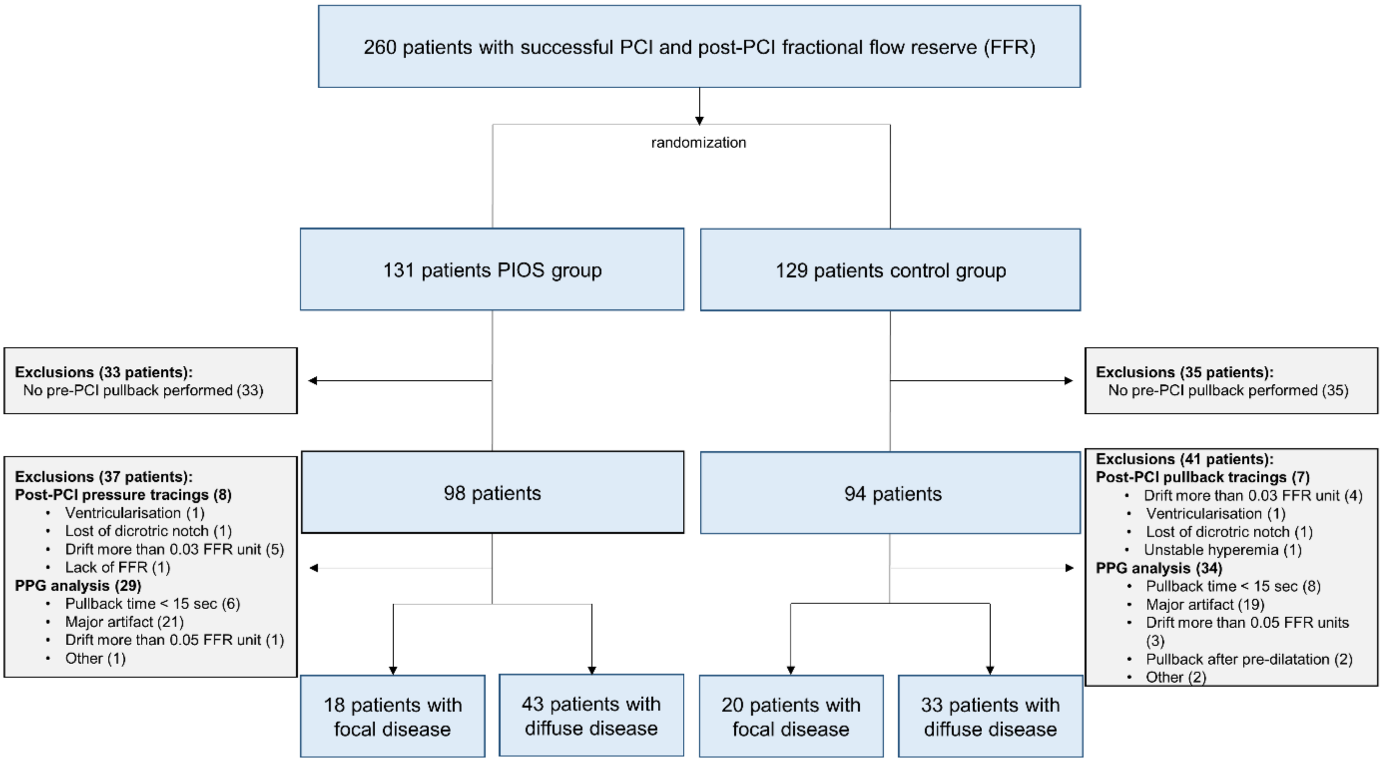

Figure S2. Study flowchart. Focal is defined as PPG ≥ 0.74 and diffuse as PPG < 0.74. FFR = fractional flow reserve; PCI = percutaneous coronary intervention; PIOS = physiology-guided incremental optimisation strategy; PPG = pullback pressure gradient;

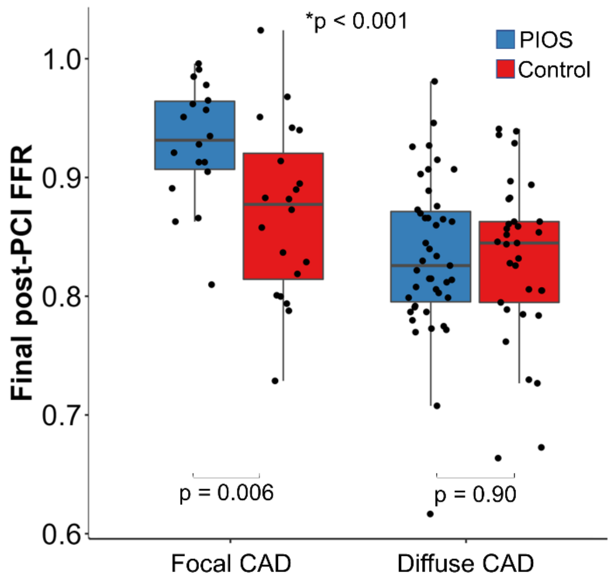

Figure S3. Final post-PCI fractional flow reserve (FFR) stratified by randomization arm and PPG defined focal or diffuse disease.

There was a significant difference in final post-PCI FFR between the focal PIOS, diffuse PIOS, focal control, and diffuse control groups. (0.93±0.05 focal PIOS vs 0.87±0.07 focal controls vs 0.83±0.07 diffuse PIOS vs 0.83±0.07 diffuse controls; p-value<0.001)
The left side panel shows focal disease stratified by randomized arm (PIOS in blue and controls in red). In patients with focal CAD, there was a significant difference in final post-PCI FFR between randomization arms. The right side panel shows diffuse disease (PIOS in blue and controls in red) with no significant difference in final post-PCI FFR between randomization arms. Focal CAD defined as PPG ≥ 0.74. *Focal CAD PIOS vs. Diffuse CAD PIOS vs. Focal CAD Controls vs. Diffuse CAD Controls group.
CAD = coronary artery disease; FFR = fractional flow reserve; PCI = percutaneous coronary intervention; PIOS = physiology-guided incremental optimisation strategy;

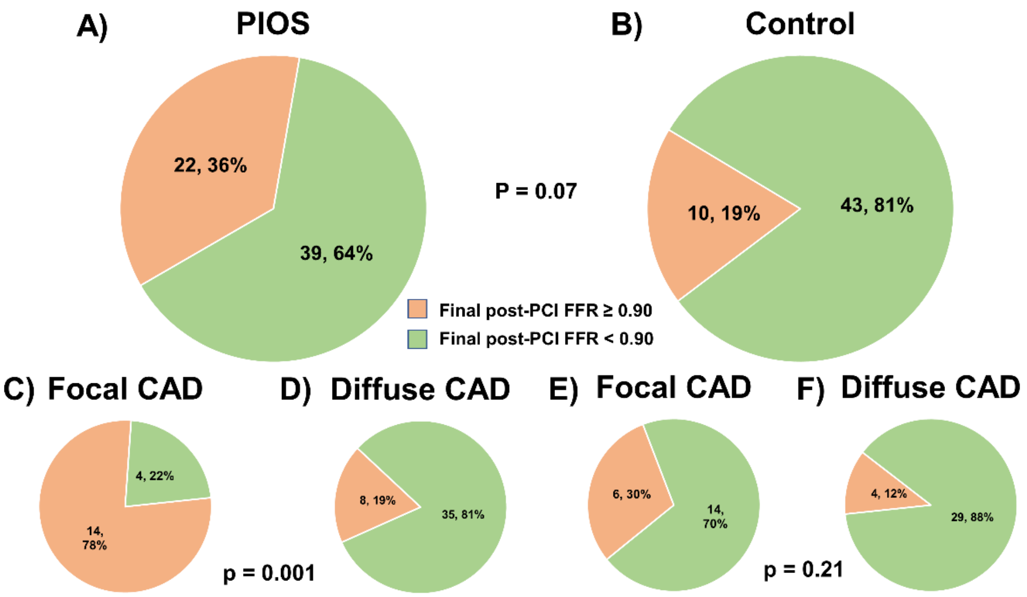

Figure S4. The rate of optimal final post-PCI FFR (≥0.90) stratified by randomization arm and focal or diffuse coronary artery disease. Panel A showed the proportion of patients achieving final post-PCI FFR ≥0.90 in PIOS group. Panel B showed the proportion of patients achieving final post-PCI FFR ≥0.90 in control group. Panel C showed the proportion of patients achieving final post-PCI FFR ≥0.90 in focal PIOS group. Panel D showed the proportion of patients achieving final post-PCI FFR ≥0.90 in diffuse PIOS group. Panel E showed the proportion of patients achieving final post-PCI FFR ≥0.90 in focal control group. Panel F showed the proportion of patients achieving final post-PCI FFR ≥0.90 in diffuse control group. CAD = coronary artery disease; FFR = fractional flow reserve; PCI = percutaneous coronary intervention; PIOS = physiology-guided incremental optimisation strategy.
